# PaperBLAST: Text Mining Papers for Information about Homologs

**DOI:** 10.1128/mSystems.00039-17

**Published:** 2017-08-15

**Authors:** Morgan N. Price, Adam P. Arkin

**Affiliations:** Environmental Genomics & System Biology, Lawrence Berkeley National Lab, Berkeley, California, USA; Dalhousie University

**Keywords:** annotation, text mining

## Abstract

Large-scale genome sequencing has identified millions of protein-coding genes whose function is unknown. Many of these proteins are similar to characterized proteins from other organisms, but much of this information is missing from annotation databases and is hidden in the scientific literature. To make this information accessible, PaperBLAST uses EuropePMC to search the full text of scientific articles for references to genes. PaperBLAST also takes advantage of curated resources (Swiss-Prot, GeneRIF, and EcoCyc) that link protein sequences to scientific articles. PaperBLAST’s database includes over 700,000 scientific articles that mention over 400,000 different proteins. Given a protein of interest, PaperBLAST quickly finds similar proteins that are discussed in the literature and presents snippets of text from relevant articles or from the curators. PaperBLAST is available at http://papers.genomics.lbl.gov/.

**IMPORTANCE** With the recent explosion of genome sequencing data, there are now millions of uncharacterized proteins. If a scientist becomes interested in one of these proteins, it can be very difficult to find information as to its likely function. Often a protein whose sequence is similar, and which is likely to have a similar function, has been studied already, but this information is not available in any database. To help find articles about similar proteins, PaperBLAST searches the full text of scientific articles for protein identifiers or gene identifiers, and it links these articles to protein sequences. Then, given a protein of interest, it can quickly find similar proteins in its database by using standard software (BLAST), and it can show snippets of text from relevant papers. We hope that PaperBLAST will make it easier for biologists to predict proteins’ functions.

## Abstract (precis)

With the recent explosion of genome sequencing data, there are now millions of uncharacterized proteins. If a scientist becomes interested in one of these proteins, it can be very difficult to find information as to its likely function. Often a protein whose sequence is similar, and which is likely to have a similar function, has been studied already, but this information is not available in any database. To help find articles about similar proteins, PaperBLAST searches the full text of scientific articles for protein identifiers or gene identifiers, and it links these articles to protein sequences. Then, given a protein of interest, it can quickly find similar proteins in its database by using standard software (BLAST), and it can show snippets of text from relevant papers. We hope that PaperBLAST will make it easier for biologists to predict proteins’ functions.

## INTRODUCTION

Genome sequencing has accelerated the discovery of novel proteins far beyond the rate at which these proteins’ functions are being determined (1). Thus, to interpret genome sequences and to annotate the role of these predicted proteins, we rely on the similarity between the novel proteins and characterized ones. Proteins with over 30% similarity are likely to have similar functions (2), but to be 90% confident of the substrate of an enzyme, over 60% similarity may be required (3).

Unfortunately, the databases of protein function that are used to make annotations are far from complete. As an example, the Swiss-Prot database (4) is the largest curated resource of functional information about proteins, with experimental evidence relating to about 80,000 proteins. Nevertheless, the Swiss-Prot curators curate only 35% to 45% of new articles about protein function, and they focus on a few well-studied model organisms (5). They “do not have sufficient resources to actively curate organisms studied by smaller scientific communities” (ibid.).

As an alternative to expert curation, text-mining tools can find scientific articles that discuss a protein of interest (see, i.e., reference 6). With these tools, biologists can quickly find articles about a protein of interest and determine its function by reading the articles themselves, instead of relying on curators to do this for them. However, most of these text-mining tools have focused on model organisms and are not suitable for annotation by homology. Specifically, we are not aware of any text-mining tools that, given a protein of interest, search for information about similar proteins. There are tools that combine BLAST searches with links to articles from UniProt and GenBank (7–9), but since these tools do not search the literature, their coverage is limited.

To allow access to the literature for searches by homology, we developed the PaperBLAST website (http://papers.genomics.lbl.gov/). Given a protein identifier or a protein sequence, PaperBLAST rapidly finds similar proteins that are discussed in the literature and provides links to those proteins and to articles about them.

## RESULTS

### Examples.

While we were studying the utilization of various carbon sources by *Pseudomonas fluorescens* FW300-N2E3, we discovered that the AO353_07705 protein is required for the utilization of l-carnitine (10). As of April 2017, AO353_07705 is annotated in RefSeq (11) as “(Fe-S)-binding protein” (see NCBI accession no. WP_054594379.1) and is annotated by SEED (12) as “Predicted l-lactate dehydrogenase, Iron-sulfur cluster-binding subunit YkgF” (see GenBank accession no. FIG00138298). Neither of these annotations explained this protein’s role in carnitine utilization. Other annotation databases did not give useful results either: running InterProScan (13) or BLASTing against UniProt (4) gave similarly vague information, and KEGG (14) did not provide a prediction (see pba:PSEBR_a5225).

**FIG 1:**
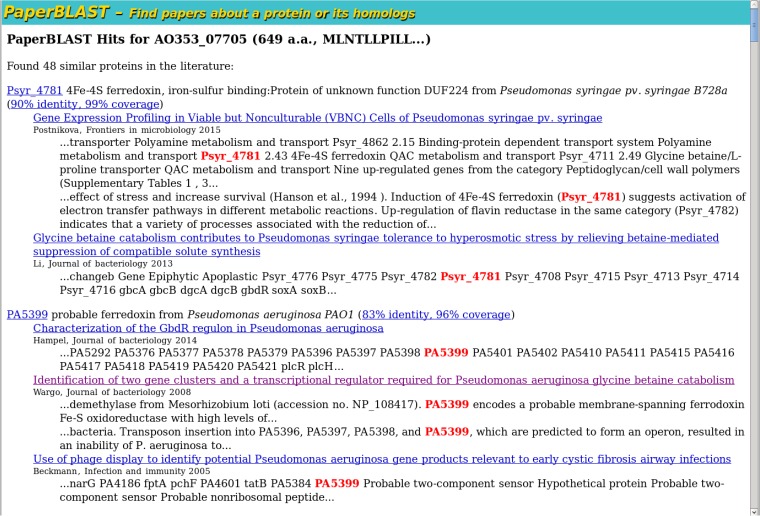
Example of PaperBLAST results. For each protein that is linked to the literature and is similar to the query protein, PaperBLAST shows a list of articles. For each article, PaperBLAST shows up to two snippets that mention the protein. a.a., amino acids.

In contrast, PaperBLAST found published information about many homologs of AO353_07705, with the two closest homologs being from other strains of *Pseudomonas* (see Fig. 1). The search took less than 3 s. The articles about the closest homolog (from *P. syringae*) discuss gene regulation and might not be functionally informative. But one of the articles about the second homolog, PA5399 from *P. aeruginosa*, reports that it is required for the catabolism of glycine betaine and for the demethylation of dimethylglycine (15). Although this level of detail is not apparent in PaperBLAST’s snippets, the snippets do mention a transposon mutant of PA5399, which makes it clear that of the five articles about the close homologs, this article is the one most likely to have functional information. Given the hypothesis that AO353_07705 is required for breaking down dimethylglycine, we can now explain the phenotype of the mutants in AO353_07705: carnitine is structurally related to glycine betaine, and some pseudomonads break down carnitine via glycine betaine and dimethylglycine (16).

This example also illustrates that information about closely related proteins may not propagate through the literature. Of the two articles that discuss the expression of the homolog from *P. syringae* (Psyr_4781), one article annotates the gene correctly (and cites reference 15). But the other article uses a vague description (“4Fe-4S ferredoxin”), even though it was published in 2015, 7 years after the function of PA5399 was reported. This oversight reflects the difficulty of finding published information about similar proteins without an automated tool.

As another example, a colleague recently identified a phenotype for a mutant in PP_0995 from *Pseudomonas putida* KT2440 and asked us for advice as to its biochemical function. As of April 2017, the only relevant information in the protein family databases (according to InterProScan and the Conserved Domain Database [17]) was that PP_0995 belonged to an uncharacterized family (PF06532 or DUF1109). SEED annotated the protein as “extracytoplasmic function alternative sigma factor,” but we were not able to identify the rationale for this annotation. UniProt, RefSeq, and KEGG had uninformative annotations (GenBank accession numbers Q88P58, NP_743156.1, and ppu:PP_0995). In contrast, PaperBLAST identified nine homologs of this protein that are discussed in the literature, including two links to functional studies. The sixth hit (41% identity) was to CC3252 or NrsF from *Caulobacter crescentus*, which is proposed to be an anti-sigma factor: overexpression of NrsF prevents the induction of the SigF regulon after dichromate stress (18). The ninth hit (33% identity) was to blr3039 or OsrA from *Bradyrhizobium japonicum*, which antagonizes the sigma factor EcfF and binds to it (19). So we predict that PP_0995 is also an anti-sigma factor and that the SEED annotation is incorrect. Consistent with this, PP_0995 is adjacent to a putative sigma factor (PP_0994), as is common for anti-sigma factors. This example illustrates that an “uncharacterized” protein family may have functional information that has not been curated, that the rationale for a protein’s annotation is often unclear, and that PaperBLAST can be useful even if no close homologs of the query protein have been studied.

### Coverage of PaperBLAST.

**FIG 2:**
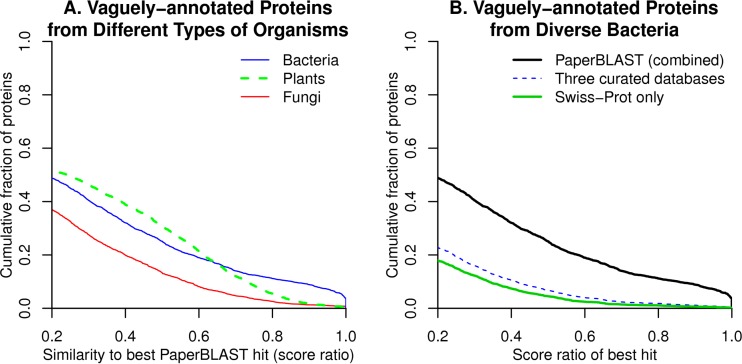
Coverage of PaperBLAST. (A) How often hypothetical proteins or other vaguely annotated proteins from different types of organisms have homologs in the PaperBLAST database with a BLAST score ratio above the given threshold. (B) How often vaguely annotated bacterial proteins have homologs in PaperBLAST, in the characterized subset of Swiss-Prot, or in any of the three curated databases that are included in PaperBLAST (the characterized subset of Swiss-Prot, GeneRIF, or EcoCyc). In both panels, only homologs with high-coverage alignments (at least 80%) were included.

To assess the odds that PaperBLAST will provide useful information for a protein, we tested it on a random sample of predicted proteins (from sequenced genomes) that do not have specific functional annotations (see Materials and Methods). We did this test separately for proteins from bacteria, from plants, and from fungi. To describe the similarity of the best hit (if any) to the query, we used the ratio of the bit scores of the alignments to the score for aligning the query against itself (the score ratio). Unlike the percentage of identity of the alignment, this takes the coverage of the alignment into account. To increase the odds that the two proteins have consistent domain content and function, we also required that the alignment cover at least 80% of the query. As shown in Fig. 2A, for 32% of bacterial proteins with vague annotations, PaperBLAST identified a homolog with a score ratio of over 0.4 that is discussed in the literature. For plant proteins and fungal proteins, the probabilities of finding a close homolog using PaperBLAST are also reasonably high (39% and 20%, respectively). These homologs are likely to have conserved functions (2). Furthermore, most of this information cannot be found in curated databases such as Swiss-Prot (Fig. 2B); for example, just 11% of vaguely annotated bacterial proteins have such close homologs (score ratio over 0.4) in the curated part of the PaperBLAST database.

However, just because a protein is discussed in the literature does not mean that there is any information about that protein’s function. So we checked a random sample of 40 of the vaguely annotated bacterial proteins that have a close homolog (ratio above 0.4) in PaperBLAST (see Data Set S1 in the supplemental material). We found that in 27 (68%) of the 40 cases, PaperBLAST found a link to genetic or biochemical data about a close homolog’s function. In 9 of the other 13 cases, the article(s) discussed gene expression or protein expression data which might not be informative as to the homolog’s function, while in 4 cases, the discussion of the homolog was based on the genome sequence only. So, we estimate that the odds of finding useful information in PaperBLAST about a vaguely annotated bacterial protein are roughly 0.32 × 0.68 = 22%.

10.1128/mSystems.00039-17.1 DATA SET S1  Analysis of 40 vaguely annotated bacterial proteins that have close homologs in PaperBLAST’s database. Download DATA SET S1, XLS file, 0.04 MB. Copyright © 2017 Price and Arkin. 2017 Price and Arkin This content is distributed under the terms of the Creative Commons Attribution 4.0 International license.

For the 27 proteins for which PaperBLAST found useful information, we also checked if a similar annotation was available using two widely used tools: SEED/RAST (12) or KEGG’s BLASTKOALA service (20). For 16 of the 27 proteins, neither resource provided an equivalent annotation (Data Set S1). In another 4 cases, RAST (but not BLASTKOALA) provided an equivalent annotation; since RAST does not provide any explanation for the basis of its annotations, the PaperBLAST results might be more useful. In summary, PaperBLAST often finds information about a protein’s function that is missing from the annotation databases.

### PaperBLAST’s database of articles and proteins.

PaperBLAST builds a database of protein sequences that are linked to scientific publications. Given this database and a protein sequence, PaperBLAST uses protein BLAST (21) to find similar sequences. This takes only a few seconds.

PaperBLAST links proteins to articles in two ways: by searching the literature for mentions of protein identifiers by using the EuropePMC database (22) and by taking advantage of manually curated resources (Swiss-Prot, GeneRIF, and EcoCyc). For an overview of how many different protein sequences are linked to articles by each resource, see Table 1. Overall, PaperBLAST contains 400,961 different protein sequences. If we cluster these sequences (23) at 80% identity, then there are 301,054 clusters. At 50% identity, there are 207,667 clusters.

**TABLE 1:** Numbers of proteins and scientific articles and links between them in PaperBLAST’s database

Source	No. of proteins^a^	No. of papers	No. of links	No. of links with a text snippet(s)
EuropePMC	315,579	73,542	639,550	613,726
Swiss-Prot^b^	79,388	27,453	38,342	
GeneRIF	77,836	662,069	1,038,801	
EcoCyc^c^	3,923	11,143	22,769	
				
Total^d^	400,961	748,450	1,721,795	

^a^ Proteins with different identifiers but with the same sequence are counted only once.

^b^ The count of proteins for Swiss-Prot includes some proteins that were linked to experimental evidence but that were not linked to articles about the protein’s function (see Materials and Methods).

^c^ The count of proteins for EcoCyc does not include proteins that are not linked to any scientific articles (even though these are included in PaperBLAST’s database).

^d^ The total is less than the sum of the parts due to overlap of data sources.

EuropePMC indexes the full texts of 4.0 million articles and the abstracts of 26.9 million articles (http://europepmc.org/contentrss; accessed 23 February 2017). PaperBLAST sends a query to EuropePMC for each protein identifier that appears in the open-access part of EuropePMC, in a PubMed abstract, or in the 499 most-referenced genomes. These identifiers are locus tags from MicrobesOnline (24) or from RefSeq (e.g., b1234, ST1234, or ACICU_01235), RefSeq protein identifiers such as WP_000822979, or UniProt accession numbers such as P10018. We use full-text queries of the form “identifier AND genus_name” to try to ensure that the article is actually discussing the protein as opposed to something else that happens to match a protein identifier. PaperBLAST also incorporates links between open access articles and UniProt or RefSeq identifiers from the text-mining efforts and EuropePMC. Because the heuristics that EuropePMC uses are different from those that we use, this yields additional links.

PaperBLAST also incorporates manually curated information from Swiss-Prot, EcoCyc, and GeneRIF about protein function. First, PaperBLAST includes proteins in Swiss-Prot (the curated part of UniProt) if they have been studied experimentally. When a Swiss-Prot protein appears in the search results, PaperBLAST shows the manually curated annotation as well as links to articles about the protein’s function, if these were provided by the curators. Second, PaperBLAST includes every protein from the well-studied bacterium *Escherichia coli*, as annotated in EcoCyc (25). Again, PaperBLAST shows the manual annotation and a link to the articles selected by the curators. Finally, PaperBLAST incorporates the gene reference into the function (GeneRIF) NCBI database (26). GeneRIF links genes to scientific articles in PubMed, and for each link, it also provides a short summary of the article’s claim about the gene’s function. For proteins that are linked to papers by GeneRIF, PaperBLAST shows the short summary.

For each link between a protein and an article from text mining, PaperBLAST tries to select one or two snippets of text that include the protein identifier. Among these links, 96% have a snippet (Table 1). In contrast, PaperBLAST does not try to extract snippets from manually curated articles: we expect that all of these articles will be more relevant and that the curator’s summary will be more useful than an automatically generated snippet.

### Completeness of PaperBLAST.

Our impression is that EuropePMC’s coverage of recent articles about proteins is high, even for papers that are not open access, but we do not have a quantitative estimate. Some older articles are missing (for example, if the journal started depositing articles into PubMed Central in 2005, the older articles might be absent). EuropePMC still searches the abstract if it does not have access to the full text, but abstracts rarely include locus tags or other protein identifiers, so older articles are often missed. On the other hand, older articles are more likely to be curated.

To check the completeness of PaperBLAST, we asked if recently published articles that characterize a protein’s function were indexed by PaperBLAST. Among the articles that were published in the open-access journal mBio in late 2016, we identified 54 whose abstracts made claims about the functions of one or more proteins. PaperBLAST linked 15 (28%) of these articles to the key proteins via text mining. As of April 2017, none of the 54 articles had been curated. By July 2017, nine articles were linked to their key proteins by GeneRIF; one of these had also been curated by EcoCyc; and none of the 54 articles appeared in Swiss-Prot. The low rate of curation illustrates why text-mining tools are necessary. If we include the more recent curated links, then PaperBLAST can link 23 (43%) of the articles to their key proteins. In another 16 cases (30%), PaperBLAST indexed an older article that discusses the function of the key protein(s). In these cases, users should be able to find the missed articles by tracing references or by full-text searches. In the 15 remaining cases (28%), the PaperBLAST database failed to indicate that the protein has been studied experimentally. Overall, PaperBLAST indexed most of the characterized proteins.

We also examined experimentally characterized proteins from two other resources: MetaCyc version 19.5 (27) and the characterized-protein database CharProtDB (28). (These are both curated resources; they date from 2015 and 2011, respectively.) We asked how often these proteins, or a nearly identical homolog (with at least 99% identity and 90% coverage), appeared in the PaperBLAST database. We found that 75% of proteins in MetaCyc were already in PaperBLAST. Similarly, 70% of the proteins in the curated part of CharProtDB were already in PaperBLAST. (This is the part entered by CharProtDB’s curators as opposed to having been imported from Swiss-Prot or EcoCyc.) This confirms that PaperBLAST’s coverage is high, and it could be improved by incorporating additional curated resources (such as MetaCyc and CharProtDB). However, the incremental benefit of adding each additional resource is modest: for example, MetaCyc contains 2,362 proteins that do not already have nearly identical homologs in PaperBLAST.

## DISCUSSION

Although PaperBLAST can often identify functional information for a homolog of a protein of interest, it is important to consider its limitations.

First, as discussed above, many of the articles that mention a protein do not actually contain any information about that protein’s function. In particular, articles about gene expression often include tables of upregulated or downregulated genes. Because these articles mention many genes, they are overrepresented in the PaperBLAST results. PaperBLAST’s snippets for these tables consist of locus tags, numbers, and gene annotations, rather than complete sentences, which is a hint that the article might not be useful. (Also, if you were to view these articles as a Web page and search for the protein identifier, it would not appear, as the tables are rendered as images. You would need to view these articles as a PDF to find the mention of the gene.) In another common situation, the article mentions a protein that is believed to have a role in the pathway under consideration, but the experimental data concern other proteins in the pathway. These articles can still be useful if they make a convincing prediction of the protein’s function. In the future, automated text analysis might be able to determine which articles have useful information about the protein’s function.

Second, PaperBLAST relies on protein identifiers, and it does not correctly interpret gene names such as *yaaA*. This works well for non-model organisms, but some articles about proteins from model organisms are missed. The incorporation of manually curated information from Swiss-Prot, GeneRIF, and EcoCyc would usually make up for this. A related issue is that PaperBLAST relies on matching by protein identifier. There are some spurious links due to other identifiers that happen to match protein identifiers. Spurious links should be obvious from the snippets, but more-sophisticated text analysis might be able to filter them out. Also, many articles include DNA sequences such as primers, which could provide an independent check of what part of what genome is being studied (29).

Third, a significant fraction of proteins are not similar to any protein that has been studied. For example, less than half of bacterial proteins have a homolog in PaperBLAST’s database with a BLAST score ratio of 0.3 or above. In the future, high-throughput approaches will provide functional information for proteins on a larger scale, and these data sets could be integrated into PaperBLAST. For example, Fitness BLAST (http://fit.genomics.lbl.gov/images/fitblast_example.html) identifies homologs that have mutant phenotypes in a collection of ∼5,000 genome-wide mutant assays from diverse bacteria (10). Of the bacterial proteins that lack a reasonable homolog (ratio > 0.3) in PaperBLAST’s database, about 5% already have such a homolog with a mutant phenotype. To highlight these cases, a short summary of the Fitness BLAST results is shown at the bottom of the PaperBLAST results page.

Finally, although PaperBLAST often finds information about the function of a homolog of the protein of interest, there is no guarantee that the homologs have the same function, even if the proteins are very similar (3). One should carefully consider other factors such as whether the proteins are evolutionary orthologs, whether the proteins have conserved context or synteny, or whether key residues are conserved.

In conclusion, PaperBLAST can quickly find articles that are relevant to the function of a protein of interest. We hope that it will be useful to every biologist who studies non-model organisms.

## MATERIALS AND METHODS

### Building the PaperBLAST database.

The key steps in building the PaperBLAST database are selecting protein identifiers to search for in EuropePMC; finding articles in EuropePMC that mention these identifiers; selecting snippets of text from these articles; linking protein sequences to curated descriptions (and usually to scientific articles as well) by downloading and parsing data from Swiss-Prot, GeneRIF, and EcoCyc; collapsing redundant identifiers for identical sequences; and building an SQL database and BLAST database to store the information.

### Selecting protein identifiers to search for.

PaperBLAST selects potential protein identifiers from PubMed abstracts and from the full text of open-access articles in EuropePMC. Only words that are likely to be identifiers are considered: after removing trailing punctuation, they must contain only letters, digits, and underscores; they must begin with a letter; and they must end with at least three digits. These potential identifiers are compared to protein identifiers from RefSeq, MicrobesOnline, and UniProt/TReMBL. For proteins in RefSeq, PaperBLAST uses the protein_id, locus_tag, old_locus_tag, gene, and gene_synonym fields. The version number is removed from the protein_id (e.g., “XP_013851078” and not “XP_013851078.1”). For proteins in MicrobesOnline, PaperBLAST selects entries in the Synonym table with type = 1, 3, 4, or 6. In practice, these are mostly locus tags (e.g., “PA0630”). For proteins in UniProt or TReMBL, PaperBLAST uses the accession and the entry name (e.g., “Q6GZX4” and “001R_FRG3G”).

PaperBLAST also uses the locus tags (Synonym types 1, 4, and 6) of all proteins in the 499 most-referenced genomes in MicrobesOnline.

### Searching EuropePMC.

For each identifier, we use full-text queries of the form “identifier AND genus_name.” (The genus name is the first word of the organism name.) The genus name is included because a locus tag might match some other identifier. For instance, “SO1000” is the locus tag of a protein in *Shewanella oneidensis*; as of July 2017, searching EuropePMC with “SO1000” returns two spurious results, but searching with “SO1000 AND Shewanella” correctly returns no results. Also, although locus tags were intended to be unique across genomes, this is not always the case, and checking for the genus name helps to disambiguate results.

PaperBLAST uses 10 parallel threads to issue queries to EuropePMC. To avoid being blocked, each thread is limited to slightly less than 1 query every 2 s, or 4.5 queries per second in total. A recent build of PaperBLAST required processing 2.1 million queries, which took roughly 500,000 s or 5 days. This is the slowest step in building the PaperBLAST database.

PaperBLAST also incorporates EuropePMC’s text-mined terms (ftp://ftp.ebi.ac.uk/pub/databases/pmc/TextMinedTerms). This gives us additional links from UniProt accession numbers and RefSeq accession numbers to scientific articles. For RefSeq, we incorporate only the mentions of protein entries.

### Selecting snippets of text from relevant articles.

The main challenge in this step is to get access to the full text of the articles. PaperBLAST obtains the full text of open access articles as well as “author manuscript” articles from EuropePMC. Some other articles are available from the text-mining Application Programming Interfaces (APIs) of Elsevier (http://api.elsevier.com/documentation/FullTextRetrievalAPI.wadl) or crossref (http://tdmsupport.crossref.org/). Additional articles are downloaded using Maximilian Haeussler’s pubCrawl2 (https://github.com/maximilianh/pubMunch).

If the full text is available, then PaperBLAST identifies words that match the gene identifier. The gene identifier must appear as a separate word (possibly with punctuation at the end). For gene identifiers with a single letter followed by numbers, such as “c1406,” which might be more likely to have spurious hits, we also require the case of the letters in the match to be the same as that in the query (i.e., “C1406” is ignored). If the full text is available but no snippet meeting these restrictions is found, then the link between the article and the gene is suppressed because it is probably a false positive. Once the gene identifier is found, PaperBLAST selects up to 15 words before and 30 words after the identifier. PaperBLAST limits each snippet to at most 160 characters and records at most two snippets per gene per article, which may be considered fair use under copyright law.

If the full text is not available, PaperBLAST tries to use the abstract (from PubMed) to compute snippets.

### Information from manually curated databases.

PaperBLAST indexes proteins from Swiss-Prot (and shows them in the PaperBLAST results) if the Swiss-Prot curators identified experimental evidence about the protein. (This is indicated by the presence of the evidence code ECO:0000269 in the comment field.) However, not all of these Swiss-Prot entries link to scientific articles in PubMed. A few of these entries are annotated based on theses or conference abstracts (or even direct submissions from scientists to UniProt). PaperBLAST does not link to these types of articles. A more common issue is that Swiss-Prot often links experimental evidence from a paper to the protein’s expression pattern or subcellular localization, which may not be functionally informative. PaperBLAST shows only the functional parts of the comment and links to the functionally relevant articles. (Specifically, in the comment field, the “topic” that mentions the article must be one of function, catalytic activity, cofactor, enzyme regulation, disruption phenotype, or subunit structure.) After these restrictions, 48% of the Swiss-Prot entries with experimental evidence link to at least one article. Even if the article(s) is not shown on the PaperBLAST website, the information is available via a link to the UniProt page for the protein.

PaperBLAST also indexes every protein from *Escherichia coli* K-12 in EcoCyc. Although most of the EcoCyc entries include links to articles in PubMed, about 200 do not—these proteins would still appear in the PaperBLAST results if they are similar to the query.

To incorporate GeneRIF, PaperBLAST converts NCBI gene identifiers to RefSeq protein identifiers. To do this, it uses the NCBI Entrez programing utilities (https://eutils.ncbi.nlm.nih.gov/entrez/eutils/). About 84,000 gene identifiers were successfully converted to 78,000 distinct protein sequences.

### Redundant sequences.

PaperBLAST uses usearch 8.0 (http://www.drive5.com/usearch/) to identify proteins in its database that have identical sequences. Duplicate proteins are removed from the BLAST database and are shown together in the output.

We also used usearch 8.0 (with the -cluster_fast and -sort length options) to determine the number of clusters of sequences at different levels of similarity.

### Settings for BLAST.

PaperBLAST uses protein-protein blast 2.2.18 from NCBI’s BLAST+ package, with filtering of low-complexity sequences during lookup but not during alignment (the arguments -F “m S”). PaperBLAST reports hits with *E* values of <0.001, up to a limit of 250 hits.

### Testing the coverage of PaperBLAST.

To test the coverage of PaperBLAST on bacterial proteins, we used a randomly selected set of 1,643 vaguely annotated proteins from diverse bacteria that we described previously (10). Briefly, we grouped the bacterial genomes in MicrobesOnline based on their phylogenetic distance; we selected one genome at random from each group of closely related genomes; and we selected 5 proteins at random from each genome. These proteins’ annotations were considered vague if they included the word “family” or “domain”; if they included the phrase “related protein” or “transporter related”; or if the entire annotation matched entries in a list of common nonspecific annotations such as “hypothetical protein,” “transcriptional regulator,” and “ABC transporter” (see the Materials and Methods section of reference 10 for the complete list); this matching was case insensitive.

To obtain a random set of fungal proteins, we randomly selected 0.2% of the proteins in the fungal section of RefSeq (release 81, April 2017). We then selected the fungal proteins whose description included “hypothetical,” “family,” or “domain,” which left a test set of 3,168 proteins. To obtain a random set of plant proteins, we randomly selected 1% of proteins from a random set of 20 genomes from Ensembl Plants release 34 (ftp://ftp.ensemblgenomes.org/pub/plants/release-34/fasta/). These genomes included five different species of the genus *Oryza*; the other 15 genomes were from unique genera. We then selected proteins whose description included “uncharacterized,” “hypothetical,” “family,” or “domain,” leaving a test set of 2,256 plant proteins.

All of these sequences were compared to sequences in the PaperBLAST database using protein-protein BLAST with *E* values of <10^−5^, with filtering of low-complexity sequences performed during lookup but not during alignment. They were also compared to themselves to allow computation of the score ratios. A few proteins that were too short to have a significant self-hit were excluded from the analysis.

To test the utility of the PaperBLAST results for these proteins, we randomly selected 40 of the vaguely annotated bacterial proteins that had PaperBLAST hits (as described in Results). We ran RAST and BLASTKOALA on these proteins on 28 June 2017. For BLASTKOALA, we used the “genus_prokaryotes” database and we specified that we were providing bacterial proteins.

### Testing the completeness of PaperBLAST.

We used papers from the September/October 2016 and November/December 2016 issues of mBio (http://mbio.asm.org/content/7/5.toc; http://mbio.asm.org/content/7/6.toc). We examined the abstract of every “Research Article” in both issues and of every “Observation” in the November/December issue. We selected abstracts that made claims about the functions of specific proteins. Abstracts that focused on describing a cellular process or on the biochemical details of a protein whose function is already characterized were ignored. For each of the 54 remaining abstracts, we selected the key proteins (usually just one or two proteins) about the functions of which the abstract made claims. We then checked if the April 2017 release of PaperBLAST linked these articles to the key proteins. If one or more of the key proteins were not linked, we checked if PaperBLAST linked these proteins to an older article that had information about the protein’s function. We also checked if UniProt, GeneRIF, or EcoCyc had curated these 54 articles.

For the 15 articles whose key proteins did not appear in PaperBLAST (even via an older paper), we examined the article to see if it could have been handled automatically. In general, these articles use short gene names such as AMA1, acrD, or AmpDh3 that are not unique across organisms and are difficult to handle automatically. Four of the articles provided primer sequences (usually in a supplementary table) that could probably be used to link to specific genes (29). Four of the articles used identifiers that PaperBLAST does not currently handle: two articles provided GenBank accession numbers for nucleotide sequences (which PaperBLAST ignores) that contain only the key proteins; one article provided a locus tag that does not appear in RefSeq (and hence is not understood by PaperBLAST) but that is indexed by UniProt; and one article included locus tags within more-complex descriptions of the mutants that were studied (i.e., “CAGL0H03487gΔ::NAT1”). Also, for one of the articles that included primer sequences, a unique identifier for the key protein was provided in a supplemental table, but the table also included identifiers for many homologs that were not studied experimentally.

For the second test of the completeness of PaperBLAST, which relied on curated databases that were not incorporated into PaperBLAST, we used MetaCyc version 19.5 (from October 2015). In MetaCyc, the proteins.dat file links to 9,369 different UniProt entries. (There are also another 236 UniProt identifiers that are no longer current and which we ignored.) We also used CharProtDB, which we downloaded in April 2017 (but the most recent entry in CharProtDB was from 2011). In CharProtDB, the “curated” portion (which is unique to CharProtDB) contained 7,636 sequences. We compared these sequences to the PaperBLAST database using BLAST.

### Data availability.

The code for PaperBLAST is available at https://github.com/morgannprice/PaperBLAST.

All analyses were conducted with the PaperBLAST database from April 2017, which is available at https://doi.org/10.6084/m9.figshare.4836407. It is based on searches of EuropePMC that were performed on or before 26 March 2017.
